# Anomaly prediction of CT equipment based on IoMT data

**DOI:** 10.1186/s12911-023-02267-4

**Published:** 2023-08-25

**Authors:** Changxi Wang, Qilin Liu, Haopeng Zhou, Tong Wu, Haowen Liu, Jin Huang, Yixuan Zhuo, Zhenlin Li, Kang Li

**Affiliations:** 1 Medical Equipment Innovation Research Center, Biomedical Big Data Center, Med-X Center for Informatics, West China Hospital, Sichuan University, Chengdu, 610041 China; 2 Sichuan University - Pittsburgh Institute, Sichuan University, Chengdu, 610207 China; 3 College of Electrical Engineering, Sichuan University, Chengdu, 610065 China; 4 Department of Radiology, West China Hospital, Sichuan University, Chengdu, 610041 China

**Keywords:** Anomaly prediction, CT equipment, Internet of Medical Things, Multivariate time series Classification, Maintenance strategy

## Abstract

**Background:**

Large-scale medical equipment, which is extensively implemented in medical services, is of vital importance for diagnosis but vulnerable to various anomalies and failures. Most hospitals that conduct regular maintenance have been suffering from medical equipment-related incidents for years. Currently, the Internet of Medical Things (IoMT) has emerged as a crucial tool in monitoring the real-time status of the medical equipment. In this paper, we develop an IoMT system of Computed Tomography (CT) equipment in the West China Hospital, Sichuan University and collected the system status time-series data. Novel multivariate time-series classification models and frameworks are proposed to predict the anomalies of CT equipment. The important features that are closely related to the equipment anomalies are identified with the model.

**Methods:**

We extracted the real-time CT equipment status time-series data of 11 equipment between May 19, 2020 and May 19, 2021 from the IoMT, which includes the equipment oil temperature, anode voltage, etc. The arcs are identified as labels of anomalies due to their relationship with decreased imaging quality and CT equipment failures. To improve prediction accuracy, the statistics and transformations of the raw historical time-series data segment in the sliding time window are used to construct new features. Due to the particularity of time-series data, two frameworks are proposed for splitting the training and test sets. Then the Decision Tree, Support Vector Machine, Logistic Regression, Naive Bayesian, and K-Nearest Neighbor classification models are used to classify the system status. We also compare our model to state-of-the-art models.

**Results:**

The results show that the anomaly prediction accuracy and recall of our method are 79% and 77%, respectively. The oil temperature and anode voltage are identified as the decisive features that may lead to anomalies. The proposed model outperforms the others when predicting the anomalies of the CT equipment based on our dataset.

**Conclusions:**

The proposed method could predict the state of CT equipment and be used as a reference for practical maintenance, where unexpected anomalies of medical equipment could be reduced. It also brings new insights into how to handle non-uniform and imbalanced time series data in practical cases.

## Background

Currently, various medical equipment has been extensively implemented in all aspects of medical services, including disease diagnosis, patient condition monitoring and rehabilitation. Particularly, the large-scale digital radiology equipment such as Computed Tomography (CT), allowing for clear cross-sectional images of internal organs through X-rays, is of vital importance for medical facilities to treat patients. However, the CT equipment, which embeds sophisticated operating systems, is vulnerable to various types of damages during its operation. Anomalies such as failures of components and system outage, which occur unexpectedly during the equipment operation, have long plagued the hospitals as a problem. The equipment anomalies could result in low quality radiographic images, unexpected delays in patient care, costly maintenance services, and even serious patient incidents. According to the Joint Commission (TJC) [1], the safety accidents such as premature deaths, severe injuries and disability accidents, are closely related to the medical equipment failures [2]. It was reported that there were a total of 176 medical equipment-related incidents in the US, accounting for 2.9% of the total number of 6093 activities collected from 8 hospitals during the period 2004–2011 [3]. Therefore, medical facilities such as hospitals and healthcare organizations must ensure high-level reliability of medical equipment to avoid operation disruptions and guarantee the patients’ safety.

To date, the maintenance strategies including Corrective Maintenance (CM) [4], Preventive Maintenance (PvM) [5–10] and Predictive Maintenance (PdM) [11–14], etc. have been widely applied to various fields such as mechanical engineering [15, 16], nuclear engineering [17], management science [18, 19], etc., which greatly improved the management level of those systems. However, the applications of maintenance models have not been thoroughly addressed on the medical equipment. Generally, most medical facilities perform equipment maintenance by following the manufacturer’s recommendations. The manufacturer establishes maintenance schedules and provides maintenance guidance for the equipment. This type of routine maintenance scheduling does improve the reliability and reduces the failure risks of medical equipment to some extent, but fails to predict and avoid the anomalies or sudden failures [20].

As various monitoring tools and technologies have been developed in the last few decades, it was announced that the combination of preventive maintenance with monitoring data along with data analysis techniques would be the appropriate approach to predict equipment anomalies [21]. The Internet of Things (IoT), which integrates the status information of machine components through the Internet, has emerged as a crucial technology to monitor the real-time status of targeted equipment [22]. Particularly, the Internet of Medical Things (IoMT), which obtains the real-time healthcare data from wearable devices and sensors [23], has received extensive attention. Currently, the development of IoMT is still at its early stage and most of the existing IoMT systems are focusing on improving the level of diagnosis related to the human body, rather than the medical equipment [24].

Many supervised ML algorithms have been applied to PdM, including Support Vector Machines (SVM) [25, 26], Ensemble Learning (EL) [27], and Deep Learning (DL) [28, 29] etc. However, these models are limited as follows. The data-driven model based on DL has high performance, but there are still many problems when dealing with small data, and it requires excessive time for training. The data-driven model based on EL also performed well, but its computation is still time-consuming. In addition, the current models based on SVM and EL lack time dependence. The current state of the equipment is affected by the state of the past period. Therefore, it is inappropriate to consider only a single record or record that is only in a relatively short time for each anomaly observation. To dealing with small data in practical application, building a time-dependent ML-based data-driven model with interpretability is necessary.

**Fig. 1:**
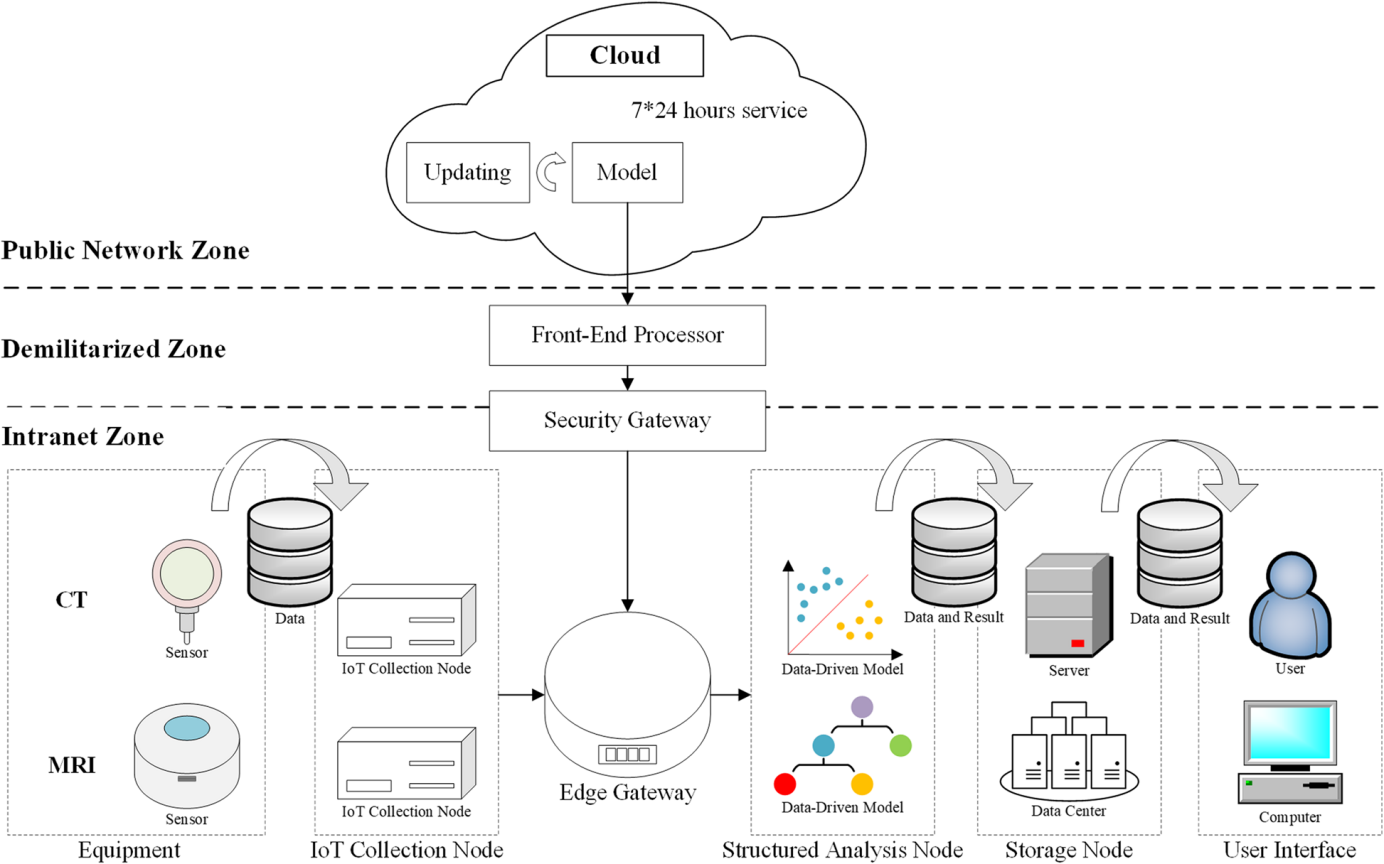
The IoMT architecture of West China Hospital

The IoMT architecture of West China Hospital is a service architecture based on Edge Computing, as shown in Fig. 1. The most significant improvement of this architecture is the use of the locally deployed Structure Analysis Node to complete the computing tasks which were originally performed by the Cloud Computing. Computing, storage, applications, communications, and other services are all deployed locally, which can ensure information security and faster network response. The data is managed by the Medical Engineering Department of West China Hospital and stored in the Data Center. The data can be accessed under strict scrutiny. Besides, data transfer, storage, model download and update from the cloud are also conducted automatically rather than manually. The involved protocols include FTP, SSH, SMB, HTTP, and HTTPS. The details of this architecture are as follows:

A) Medical equipment usually has sufficient sensors (including temperature sensor, voltage sensor, etc.) in the original design and the sensor data are stored in the log files. The IoMT of West China Hospital collect log files from equipment through IoT Collection Nodes and sends them to Structured Analysis Node. Data-driven models obtained from the Cloud in Structured Analysis Nodes are used to analyze the data extracted from the server (deployed in the Data Center). The data and the analyzed results are stored on the server and displayed to the end user through the user interface. The data transmission uses two-way financial encryption transmission based on AES 256-bit, which effectively reduces the possibility of information leakage, interception, or tampering during data transmission. The data collection box in the IoT Collection Nodes has passed the China Compulsory Certification, the ISO9001 certification, and the ISO27001 certification and conforms to China’s national confidentiality standards.
B) The data-driven model is continuously optimized through the statistical results of public computing services to ensure the improvement of model accuracy. Besides, the Cloud provides 7 × 24 h services for equipment to provide real-time updated models. Before the analysis, the Structured Analysis Node sends a model (such as anomaly prediction model) request connection through the Security Gateway and Front-End Processor to obtain the updated model from the cloud. The deployment of the Security Gateway and Front-End Processor greatly protects information security. The Front-End Processor, which has information interaction with the server, is deployed in the Demilitarized Zone of the hospital. To ensure data security, the Public Network Zone and Intranet Zone only conduct limited necessary path information exchange approved by the information regulatory authority.

In this paper, we develop a data-driven model to predict CT equipment anomalies based on the real-time status data of CT equipment obtained from the IoMT in West China Hospital, Sichuan University. The CT status parameters that are related to its condition, such as oil temperature, anode voltage, daily arcing time and daily scan time, etc. are continuously monitored. Our research can significantly minimize the stagnation and losses and improve the maintenance management. To the best knowledge of the authors, this is the first time that a sophisticated IoMT on large-scale medical equipment is developed and meanwhile, to be applied to investigate the anomaly of the medical equipment. Meanwhile, it is fairly new to combine the state-of-art machine learning models with the advanced monitoring tools in the medical field.

The rest of this paper is organized as follows: Sect. " The CT equipment and IoMT data" describes a typical CT system and the dataset we obtained from the IoMT of the West China Hospital, Sichuan University; Sect. " Methods" introduces our research procedures; Sect. " Results and analysis" shows the results; conclusions, discussions and future research directions are given in Sect. " Discussion".

## The CT equipment and IoMT data

### The CT Equipment

**Fig. 2:**
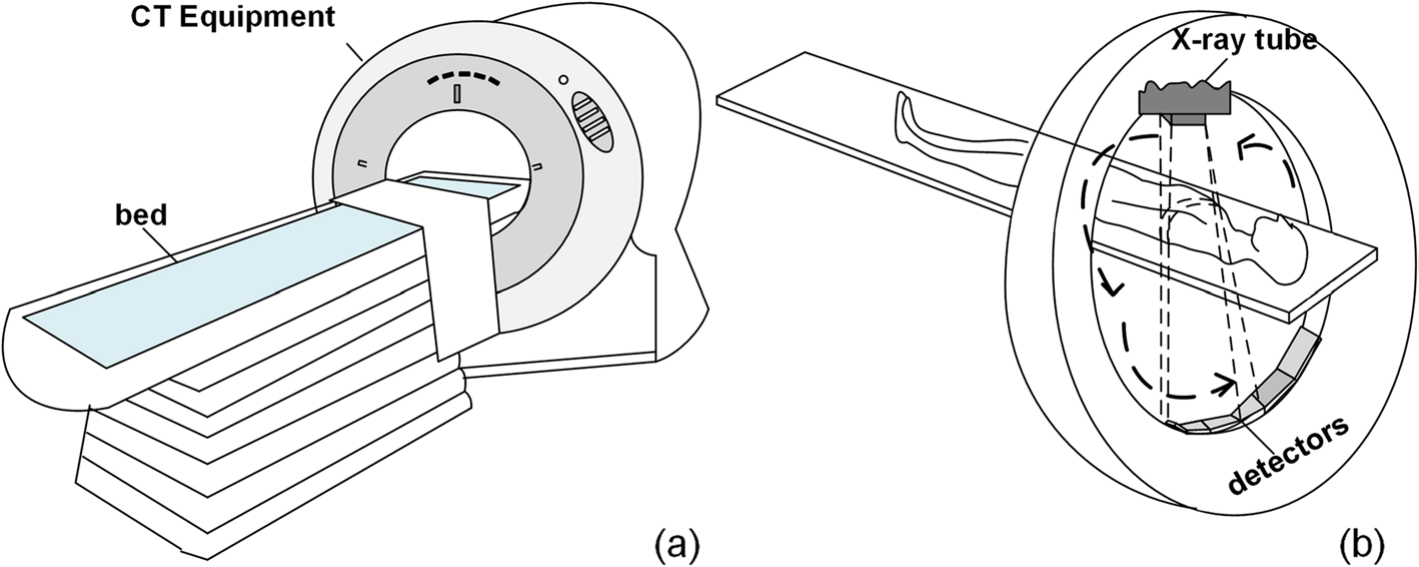
A typical CT equipment and a scan process

Figure 2 shows the diagram of a typical CT equipment. CT equipment usually operates at the maximum power rate in order to obtain the best image quality [30]. In a scan process, the fan-shaped beam of X-rays emitted from the CT tube passes through the patient onto a number of digital detectors, which receive the X-rays and convert them into medical images. During this process, the X-ray tube generates a large amount of heat while only about 1% of electrical energy is effectively converted into X-rays [31].

**Fig. 3:**
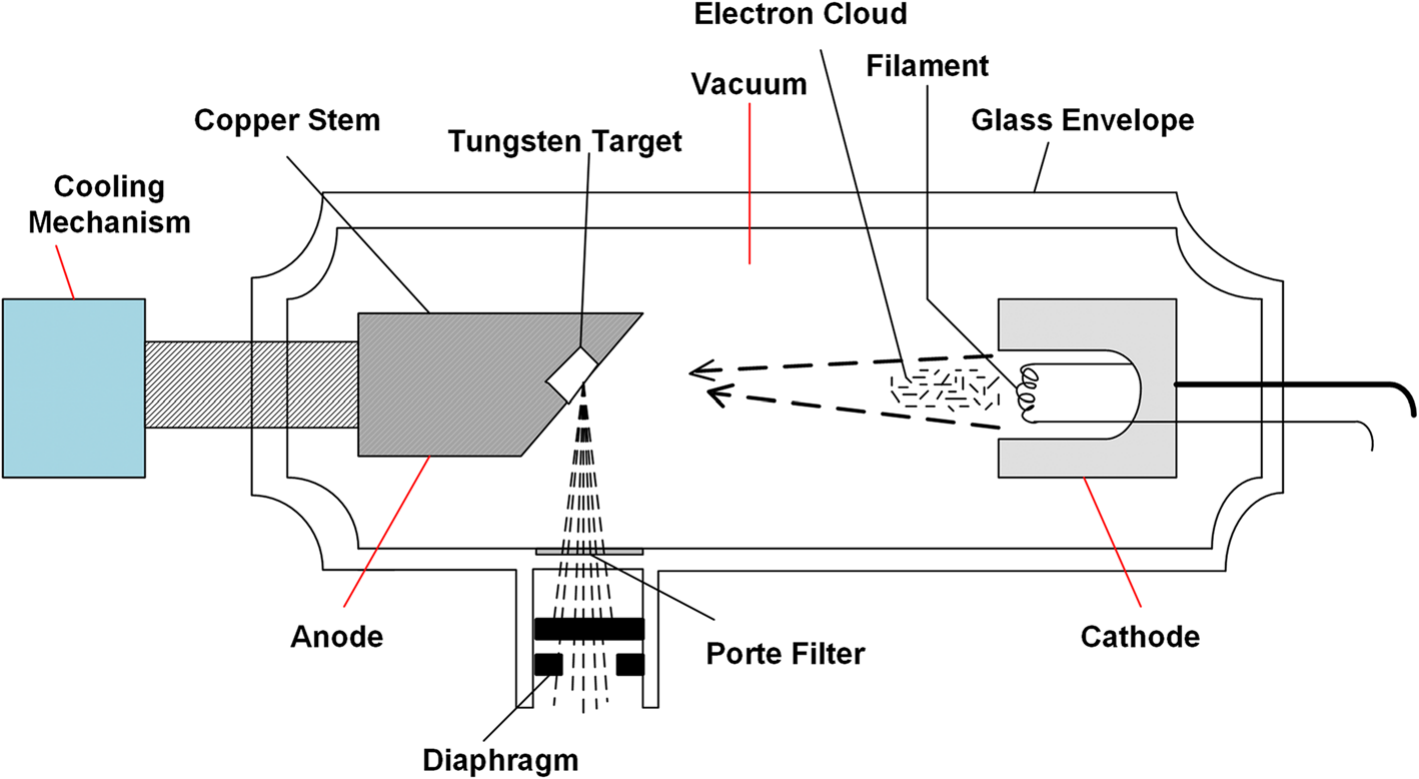
The working mechanism of a CT tube

The overheating caused by varies reason such as overload operation of the CT tube may cause damage [31].Firstly, the cathode and anode evaporate at high temperature, resulting in glass metallization, which will lead to arcing generation [32]. Secondly, the sublimation of tungsten wire caused by high temperature will cause tungsten particles to be emitted into the vacuum area to form impurities [30, 33]. The class cracks caused by high temperatures during exposure may allow air to enter the vacuum tube [30, 32]. However, the X-ray tube that works at high voltages requires a high-vacuum environment inside the tube. Arcing occurs when the required vacuum environment is broken and a conductive bridge between the cathode and the impurities is formed [34]. As shown in Fig. 3, the cooling system is used to dissipate heat, and the cooling oil can reflect the tube temperature. Besides, arcing may be generated when the operation of the equipment is at the maximum power rate for a relatively long time in a fixed period [30]. In addition, poor sealing of the vacuum tube will cause air to enter slowly and damage the vacuum environment as the tube age increases. Moreover, the voltage and current of the X-ray tube are also key factors that are related to the arcing of the tube [32].

Arcing not only could result in low-quality radiographic images, but also is closely related to the unstable performance of CT or breakdown of the tube [34, 35]. Arcing can cause artifacts that are seen as near-parallel and equidistant streak patterns or “horizontal” hypodense bands in images, which can reduce the quality of images and affect the clinical diagnosis [33, 35]. In addition, although various anomalies of X-ray tubes have been discussed in the literature, tube arcing is generally considered as the most typical and dangerous early sign of a CT equipment failure, indicating the end of the life expectancy of the X-ray tube [30, 32]. X-ray tubes are very sensitive to electronic breakdown caused by arcing, which may directly damage the tube insulation layer, thus causing irreversible damage [34]. The tube damage will make the equipment unusable and even cause the patient to die due to exposure to radiation. Therefore, arcing is worthy of attention as it is closely related to image quality and CT failure.

### Data Description

In this study, the continuously monitored real-time CT equipment status data from the IoMT of the West China Hospital is used to predict the anomaly of the CT equipment. This dataset contains the operational status data of 11 CT equipment. The features of the dataset include: Oil Temperature ($OT$), Anode Voltage ($AV$), Cumulative Tube Scanning Time ($TST$) and Cumulative Consumption of the Electrical Energy ($CE$). The appearance of arcing in the tube, which is closely related to image quality and CT failure as previously explained in Sect. " The CT Equipment", is treated as labels. As the arcing data of only 3 CT equipment are available, we only consider the data of these 3 equipment (CT1, CT2, CT3) in this project. The details of the dataset are shown in Table 1. As shown in the Gantt chart in the table, missing data appears from time to time in the dataset due to IoMT system malfunction. There are a total of 33 observations in the arcing class, which is significantly smaller than that of 733 in the non-arcing class, the dataset is imbalanced. This will be considered in the model development.

**Table 1:**
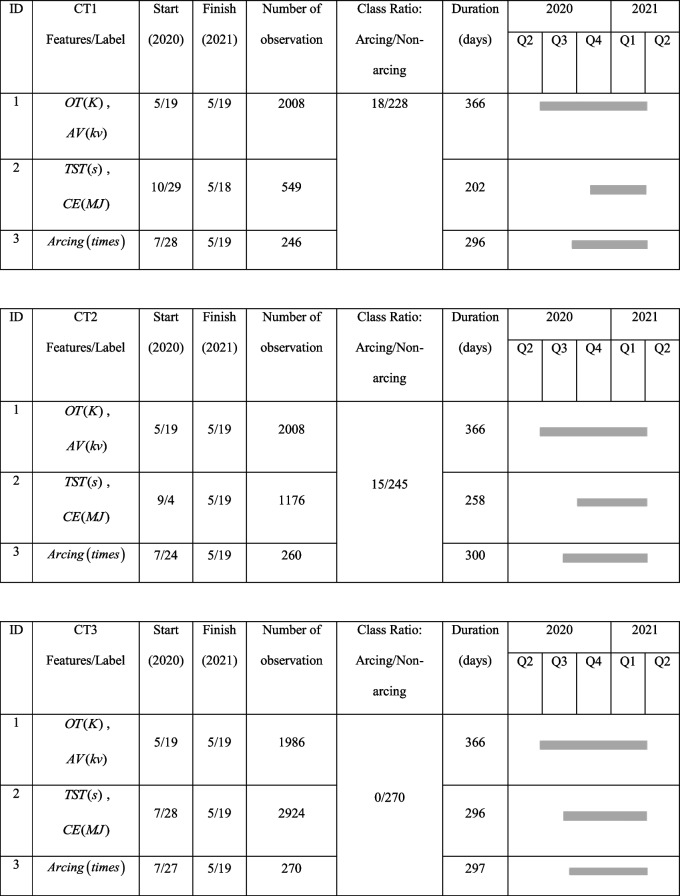
The details of the CT dataset from the IoMT

## Methods

### Data preprocessing and features construction

**Fig. 4:**
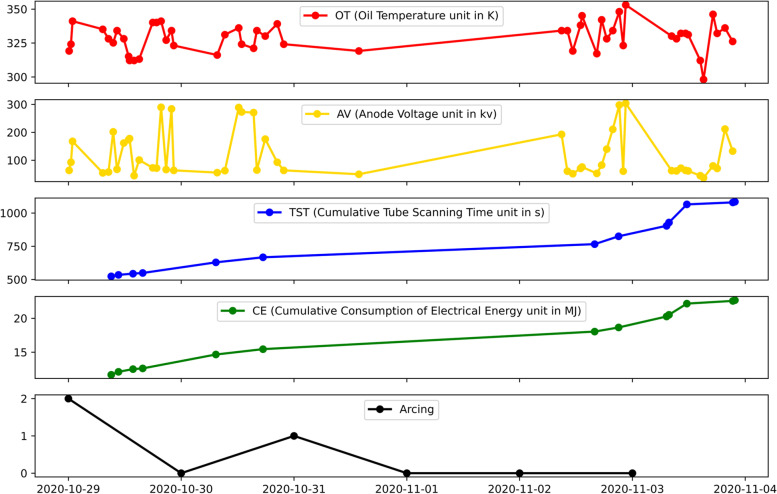
An example of the raw time series data of a CT equipment

As shown in Fig. 4, the raw observations from different IoMT sensors were obtained at a non-uniform frequency. We average the original observations of each sensor for that day and get the daily average data. In this way, the following features are obtained: the Daily Average Oil Temperature ($AOT$), Daily Average Anode Voltage ($AAV$), Daily Average Cumulative Tube Scanning Time ($ATST$), and Daily Average Cumulative Consumption of Electrical Energy ($ACE$).

To further distinguish the arcing generation from other situations, new features are constructed and considered in the model. The new features including Daily Tube Scanning Time ($DTST$) and Daily Consumption of Electrical Energy ($DCE$) are obtained by taking the first-order difference of $TST$ and $CE$ data. In addition, as the CT tube anomaly is closely related to the equipment operating current (tube current is considered here, which is direct current [36]), the equipment current is obtained as a new feature Current ($I$) based on the following equation:

1 $$I{ = }\frac{DCE}{{AAV \cdot DTST}}$$

The CT equipment health state is also related to its idle time due to the cold emission phenomenon [9] which leads to the ionization and arcing inside the idle X-ray tube. Based on this phenomenon, the new feature $IDLE$ is created, which indicates whether the X-ray tube is idled in the past $n$ days. Besides, in order to improve the accuracy of the model, the derivation of the $AOT$, $AAV$, $DTST$, $DCE$, and $I$ data are obtained and denoted as $AOT_{d}$, $AAV_{d}$, $DTST_{d}$, $DCE_{d}$, $I_{d}$ [37].

The Sliding Window algorithm is a method that has been widely implemented to predict future values, which constructs new features using the historical data in the previous days. In constructing the sliding window features, we follow these two rules:

1. Extract the maximum or average values in the time window.
2. Based on (1), if the value meets a certain condition, it is marked as 1. Otherwise, it is marked as 0.

Based on 3.1, the following features are obtained as shown in Table 2. Note that the Z-Score Normalization technique is used to normalize the features when applying rule 1.

**Table 2:** Features description

Feature	Description	Window size	Time lag
$AOT_{Max}$	Maximum $AOT$ data in the time window	$n_{1}$	1
$AAV_{Max}$	Maximum $AAV$ data in the time window	$n_{2}$	1
$I_{Max}$	Maximum $I$ data in the time window	$n_{3}$	1
$IDLE$	$IDLE = 1$ if there is at least one day IoMT receives no data from the equipment in the time window. Otherwise, $IDLE = 0$	$n_{4}$	1
$AOT_{dMax}$	$AOT_{dMax} = 1$ If the maximum absolute value of $AOT_{d}$ is greater than a threshold $a$ in time window. Otherwise, $AOT_{dMax} = 0$	$n_{5}$	1
$AAV_{dMax}$	$AAV_{dMax} = 1$ If the maximum absolute value of $AAV_{d}$ is greater than a threshold $b$ in time window. Otherwise, $AAV_{dMax} = 0$	$n_{6}$	1
$I_{dMax}$	$I_{dMax} = 1$ If the maximum absolute value of $I_{d}$ is greater than a threshold $c$ in time window. Otherwise, $I_{dMax} = 0$	$n_{7}$	1
$Arcing_{w}$	$Arcing_{w} = 1$ if there is at least one day IoMT receives the observation of arcs in the tube from the equipment in the time window. Otherwise, $Arcing_{w} = 0$	$n_{8}$	1

### Training and testing dataset construction

According to Sect. " Data Preprocessing and Features Construction", the new instance is formed by extracting the statistical values of the past period and marking the labels transformed by the time window. We compare the performance metrics using fivefold cross-validation for each parameter combination to fully use data, which can guarantee the reliability of the results. Data are split into five datasets, where one of them is used as the test set and the rest are used as the training set. However, the positive instances are concentrated over a small period of several months. If an equipment is split into five datasets in chronological order, some datasets will have no positive instances. Therefore, we choose to arrange the negative instances in chronological order and split them into five datasets. For positive instances, we choose to bundle them from adjacent days and randomly split them into five datasets.

**Fig. 5:**
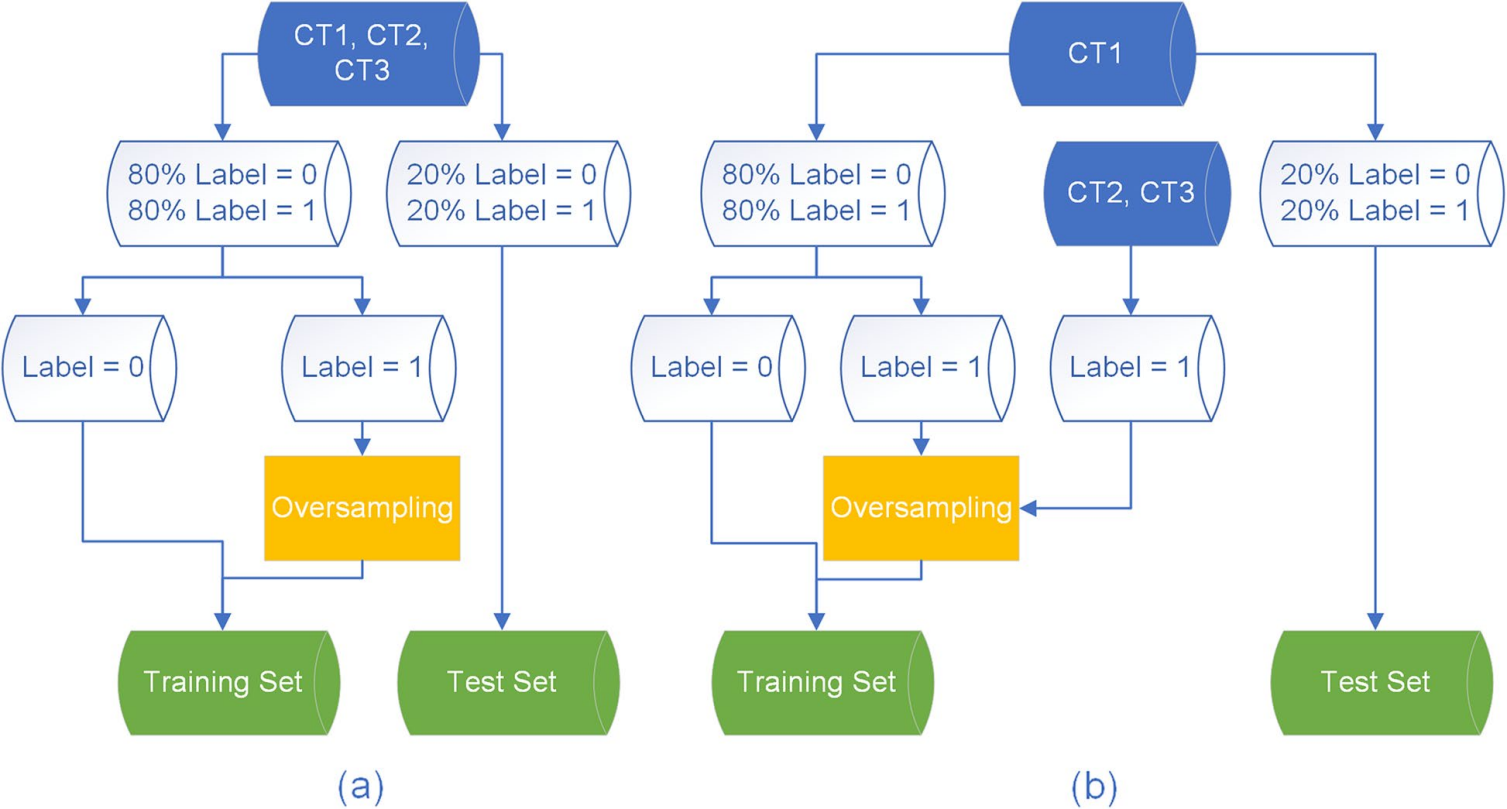
Schematic diagram of the two training and test dataset construction frameworks

As the positive observations are mainly from CT1, two frameworks are proposed to construct the training and test datasets. As shown in Fig. 5, framework 1 uses the data from all the three equipment and split it into training and test set, while framework 2 only uses data from equipment CT1, split it into the training and test set and then add the positive instances from the other two equipment training set. Random oversampling is used to increase the size of the minority class [38].

### Multivariate time series classification models

Models including Decision Tree (DT), SVM, Logistic Regression (LR), Naive Bayesian (NB), and K-Nearest Neighbor (KNN) are used in the prediction of anomalies in the multivariate time series dataset. The performance of those models are compared and the optimal one is selected. The optimal window size parameters in Table 2 are obtained, which result in the best model performance. The model is then compared with several state-of-the-art time series classification models, including BOSS [39], CIF [40], DrCIF [41], TDE [42] and DTW [43]. The model is also compared with the ML-based PdM model which lacks time dependence.

### Performance evaluation

Accuracy (Acc), Recall (Rec), Precision (Pre), and F1-score (F1) are used as the performance metrics of the classification models. Acc is calculated by Eq. (2) to reflect the overall classification ability. Rec is calculated by Eq. (3), which is the fraction of true anomalies that are predicted as anomalies. Pre is calculated by Eq. (4), which is the fraction of predicted as anomalies that are the true anomalies. F1 is calculated by Eq. (5), which is the performance metric that considers both Rec and Pre simultaneously. We also draw the receiver operating characteristic (ROC) curve and calculate the area under the curves (AUC) to evaluate the performance of the classification model.

2 $${\text{Acc = }}\frac{{\text{TP + TN}}}{{\text{TP + TN + FP + FN}}}$$

3 $${\text{Rec = }}\frac{{{\text{TP}}}}{{\text{TP + FN}}}$$

4 $${\text{Pre = }}\frac{{{\text{TP}}}}{{\text{TP + FP}}}$$

5 $${\text{F1}} = 2*\frac{{{\text{Rec}}*{\text{Pre}}}}{{{\text{Rec}} + {\text{Pre}}}}$$

where TP is true positive, TN is true negative, FP is false positive and FN is false negative.

However, the label in the training and test set has been transformed by the time window. The explanation of the performance matrix is modified according to the actual situation. For example, Acc is the probability of correctly predicting whether arcing will occur in the next few days.

## Results and analysis

**Fig. 6:**
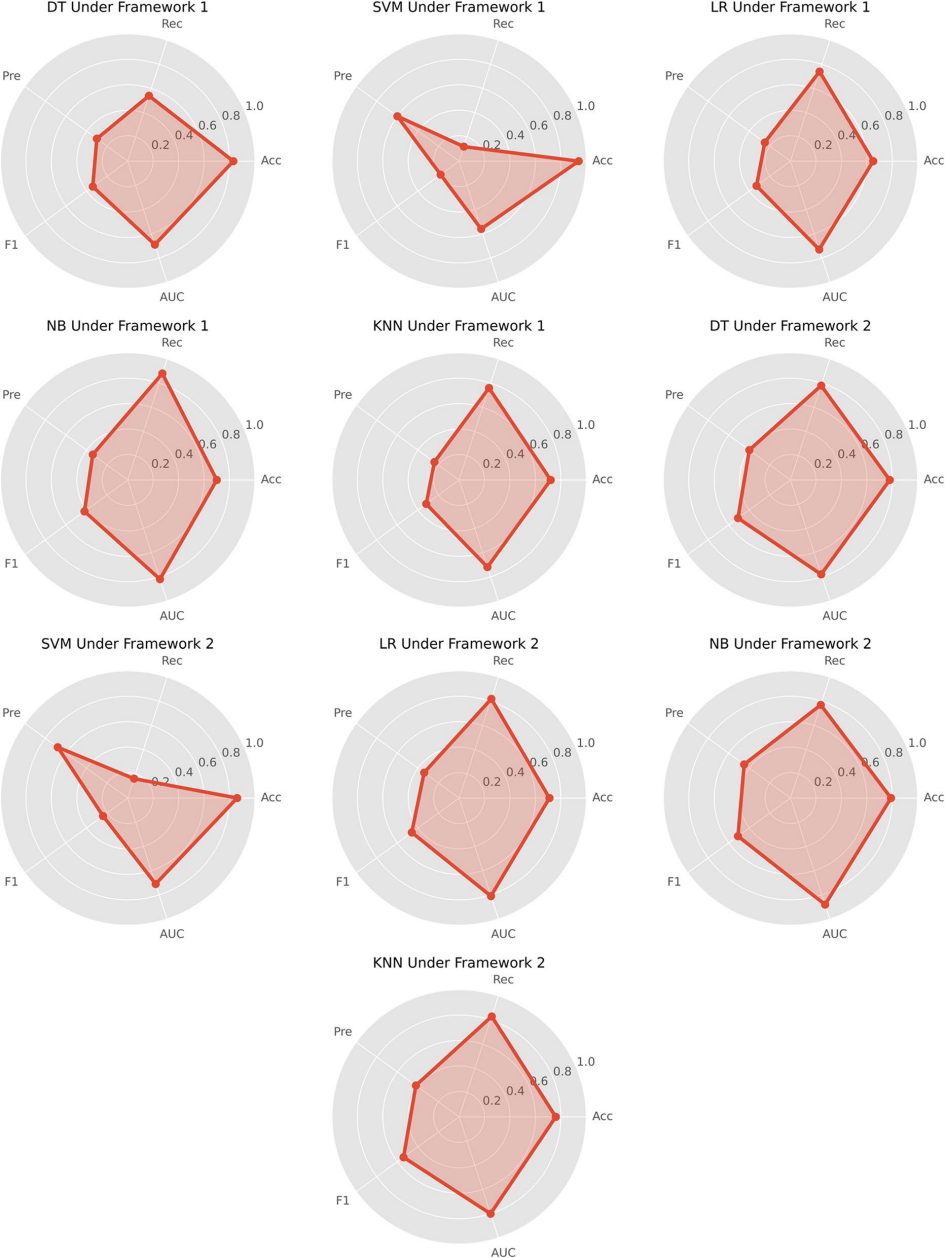
Performance metrics of the classification models

**Fig. 7:**
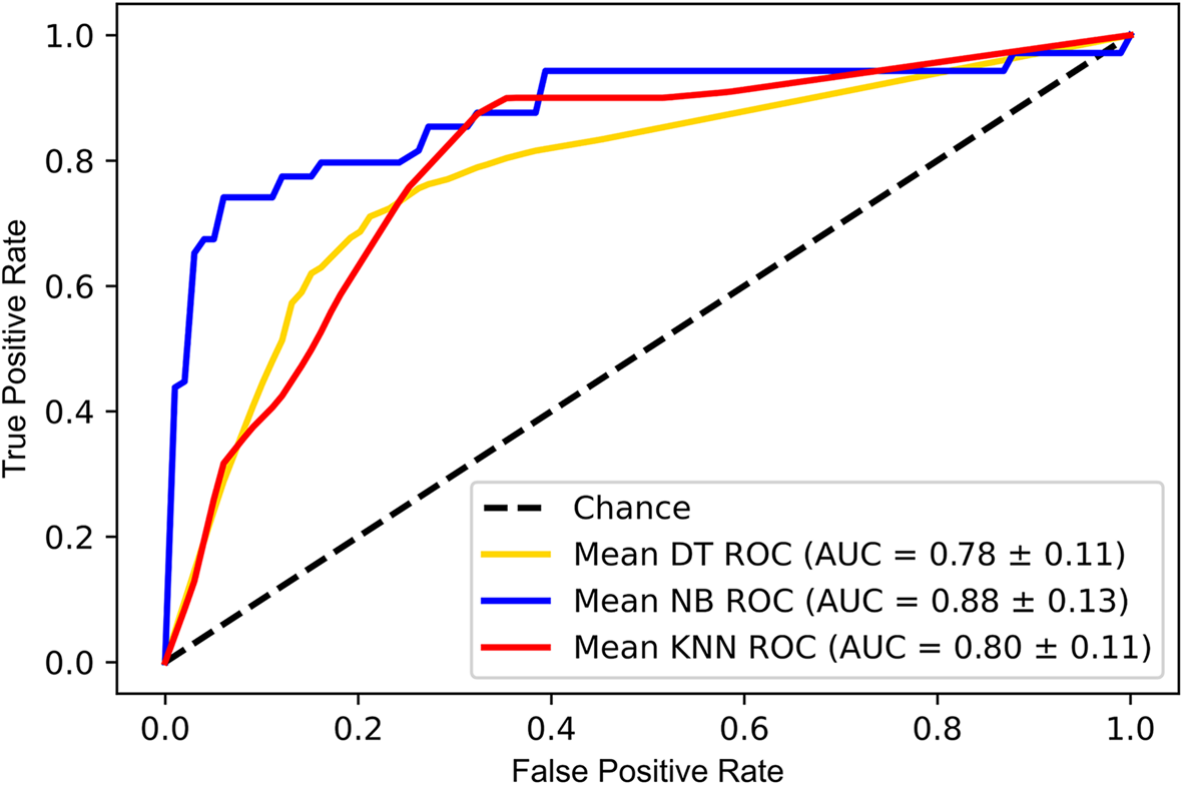
Average ROC and AUC values of the three models

The windows system with an 8-core CPU, the programming language Python (version 3.9), and the software tool Spyder (version 5.1) are used to read the data and build the model. Table 3 and Fig. 6 show the performance metrics of the 5 models under the two frameworks with the optimal parameters. The overall performance of the models under framework 2 are better than those under framework 1. Particularly, the performance metrics of NB model and KNN model under framework 2 are the best. The NB model has higher Acc, Pre, and AUC, indicating that the model has a lower false alarm rate. On the contrary, the KNN model has higher Rec and F1, indicating that the model has a higher ability to alarm on anomalies. In addition, DT models also perform well. The ROC curves and the AUC values of the three models under framework 2 are obtained as shown in Fig. 7. It is observed that the NB model, with the highest (0.88) AUC value, has the best overall classification performance. The training time of DT, NB and KNN model under framework 2 is 0.671, 0.679 and 0.837 h respectively. Table 4 shows the optimal parameters of DT, NB, KNN model under framework 2.

**Table 3:** Performance metrics of the classification models

	Models	Acc	Rec	Pre	F1	AUC
framework 1	DT	0.83	0.54	0.30	0.34	0.69
	SVM	0.94	0.12	0.60	0.18	0.56
	LR	0.65	0.74	0.25	0.33	0.73
	NB	0.70	0.88	0.34	0.42	0.82
	KNN	0.72	0.76	0.24	0.32	0.72
framework 2	DT	0.78	0.78	0.40	0.51	0.78
	SVM	0.86	0.16	0.68	0.24	0.71
	LR	0.71	0.82	0.34	0.46	0.81
	**NB**	**0.79**	**0.77**	**0.45**	**0.51**	**0.88**
	**KNN**	**0.76**	**0.83**	**0.42**	**0.54**	**0.80**

**Table 4:** The optimal model parameters

	$n_{1}$	$n_{2}$	$n_{3}$	$n_{4}$	$n_{5}$	$n_{6}$	$n_{7}$	$n_{8}$	$a$	$b$	$c$
DT	3	4	3	4	5	4	3	3	5	40	0.216
NB	5	5	5	5	5	5	3	3	5	40	0.180
KNN	5	5	4	5	4	5	4	3	5	50	0.252

The two most important features identified by the three models under framework 2 are shown in Table 5. The results show that $AOT_{Max}$ and $AAV_{Max}$ are the most important features in models DT and KNN, while $I_{dMax}$ and $AOT_{Max}$ are the most important features in model NB. This suggests that the raw features $OT$ and $AV$ are of primary importance in predicting the anomalies. Meanwhile, it also indicates that the reliability can be improved by properly operating the CT equipment, e.g., adjust the anode voltage slowly rather than quickly.

**Table 5:** The two most important features identified by the three models under framework 2

	DT	NB	KNN
1	$AOT_{Max}$	$I_{dMax}$	$AOT_{Max}$
2	$AAV_{Max}$	$AOT_{Max}$	$AAV_{Max}$

The performance metrics of the proposed model are compared with other state-of-the-art models. We apply each model to the dataset under the two frameworks as described in Fig. 5, and show the performance metrics of the one with the best performance in Table 6. It is observed that the proposed model has the best performance among all the models in this situation.

**Table 6:** The best performance metrics for models

Model	Acc	Rec	Pre	F1	AUC
BOSS	0.54	0.70	0.16	0.26	0.61
CIF	0.62	0.51	0.22	0.29	0.63
DrCIF	0.72	0.50	0.24	0.32	0.60
TDE	0.65	0.49	0.20	0.27	0.63
DTW	0.66	0.52	0.20	0.25	0.61
Our Best Model: NB	0.79	0.77	0.45	0.51	0.88
Our Best Model: KNN	0.76	0.83	0.42	0.54	0.80

The performance metrics of the proposed model were compared with the model lacking time dependence. DT, SVM, LR, NB, and KNN algorithms are used to represent ML-based models, and Random Forests (RFs) algorithms are used to represent EL-based models. However, the DL-based model is not considered here because the data size is insufficient. Two frameworks are used to train and test the performance of the time-independent models. Table 7 shows that our proposed model has higher performance in our situation.

**Table 7:** The best performance metrics for time-independent ML-based models

Model	Acc	Rec	Pre	F1	AUC
DT	0.75	0.57	0.31	0.37	0.68
SVM	0.92	0.06	0.24	0.08	0.52
LR	0.68	0.64	0.28	0.36	0.67
NB	0.71	0.41	0.26	0.28	0.60
KNN	0.64	0.75	0.28	0.39	0.69
RFs	0.74	0.63	0.35	0.42	0.70
Our Best Model: NB	0.79	0.77	0.45	0.51	0.88
Our Best Model: KNN	0.76	0.83	0.42	0.54	0.80

**Fig. 8:**
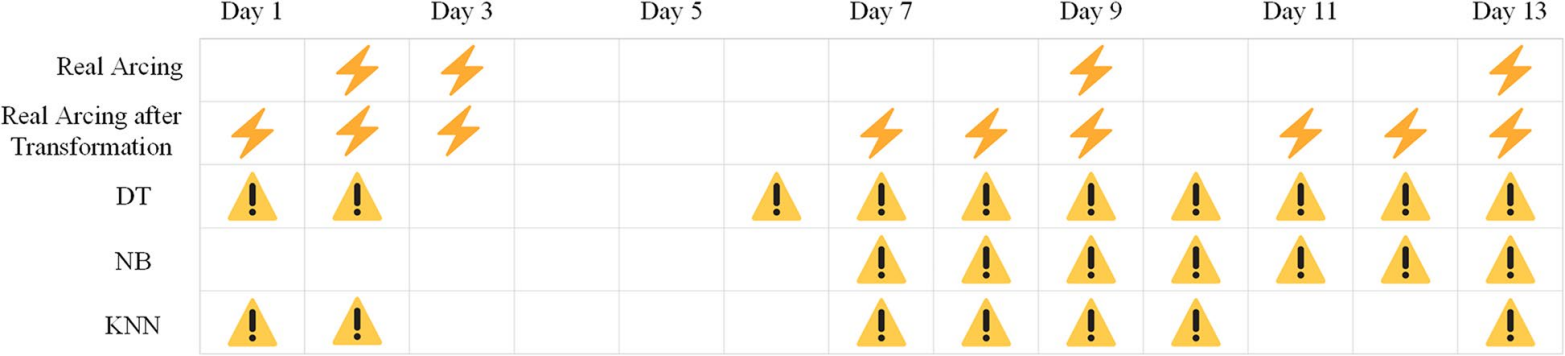
The arcing and prediction result of three models

In order to show the prediction ability of the model more intuitively, part of the data is selected as the test set, and the remaining data is used as the training set to train the model and make the prediction. Figure 8 shows the real arcing, the real arcing transformed by using the time window, and the prediction results of the three best models from December 13, 2020, to December 25, 2020 in equipment CT1. The lightning mark in the figure represents the generation of the real arcing and the real arcing transformed by using the time window. The exclamation mark in the figure represents the result predicted by the three best models using historical data. The results show that our model has the ability to predict the arcing accurately.

**Fig. 9:**
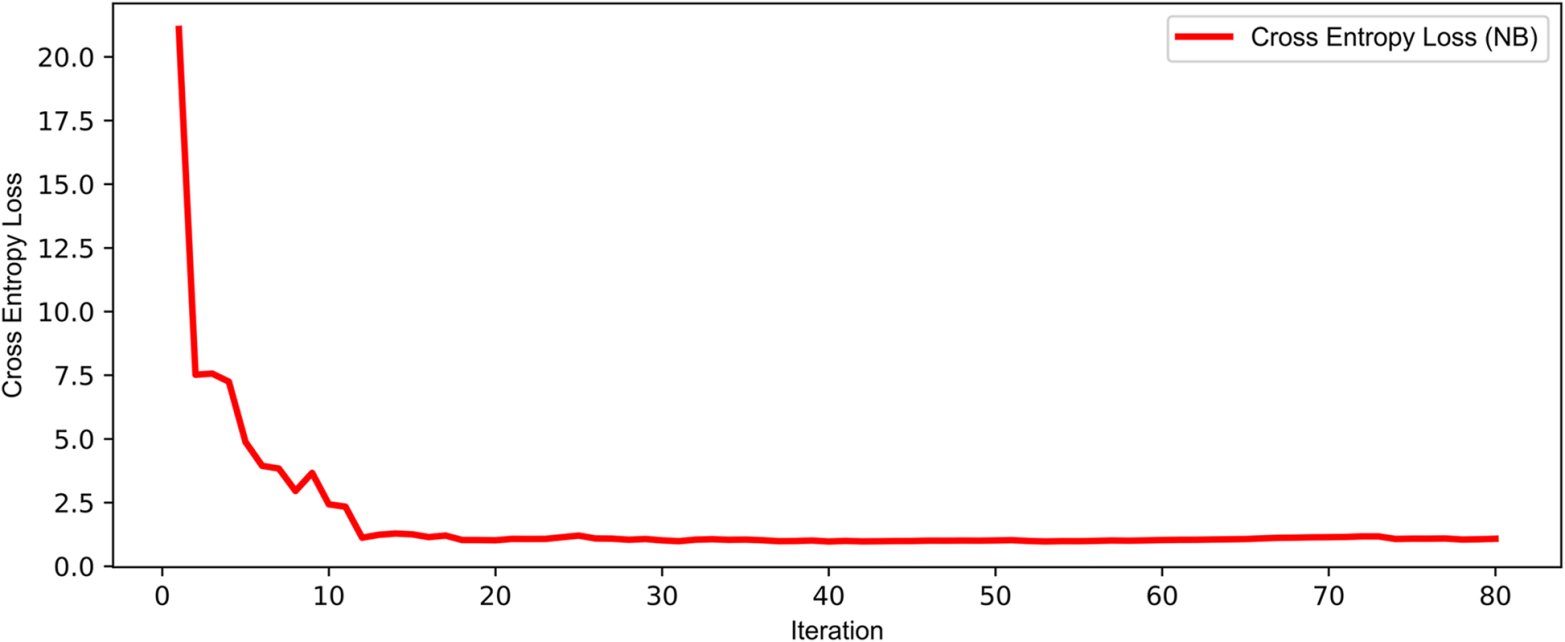
The average cross-entropy loss of each iteration in the NB model

In order to verify whether the dataset size can guarantee the model’s reliability, the training sets with different instance combinations are used to train the model and the cross-entropy loss of the test set is calculated. Firstly, the instances are divided into training and test set. Then the training set is split into several bins with equal-size of instances. In the first iteration, a bin is selected from the candidate training set as the training set of the model, and the average cross-entropy loss of the test set is calculated. For each iteration, add a bin to the training set and calculate the average cross-entropy loss of the test set again. The fivefold cross-validation is used to repeat the above step and calculate the average loss. Take the NB model under framework 2 as an example as shown in Fig. 9. It is observed that with the increase of the data size in the training set, the cross-entropy loss of the test set decreases quickly at first and then maintains at a stable state. The increase of data size stop reducing the average loss of test sets after several iterations. Therefore, it is evident that the proposed models are reliable based on the current data.

## Discussion

Given the losses of hospitals and patients that may result from the anomalies of medical equipment, predicting those anomalies in advance is of vital importance. In our work, a data-driven model that preprocesses and analyzes time series status data obtained from the IoMT is proposed to predict the anomalies of the CT equipment. Specifically, seven new features are constructed through the sliding time window based on the failure mechanism of CT equipment. Based on random oversampling, two methods of splitting training and test sets are proposed to deal with imbalanced data.

In this study, we demonstrate the applicability of classification models to the prediction of anomalies of medical equipment, which are continuously monitored by the IoMT in the hospital. Whenever the sensor collects data and stores it in IoMT, the data-driven model will use current and past data to predict whether the equipment will be an anomaly in the next few days. It is shown that the proposed model is better than existing models in this application. In practice, although large-scale medical equipment such as CT are critical for disease diagnoses and are extensively used every day, very few in-depth studies have been conducted to ensure its reliability during the operation. The ability of the regular maintenance strategies recommended by the manufacturers are limited in preventing unexpected failures. To the best knowledge of the authors, the work of this paper is a pioneering attempt to predict anomalies of large-scale medical equipment based on the IoMT data. It enables the maintenance team to estimate the reliability of equipment in real-time and make proper maintenance decisions accordingly. In the future, the data-driven methods combined with the IoT technology, which are flexible in incorporating unexpected failures of equipment, will show great potential in ensuring the reliability of equipment in the medical field.

The work has some limitations. First, although 11 CT equipment are monitored by the IoMT, the status data of only 3 CT equipment are complete and are used for model development. In the future work, more high-quality CT equipment status data should be used for model development, training and testing. Second, the feature $I$ is slightly different from the real value, because it is estimated from the $DCE$, $AAV$, and $DTST$. In the future work, sensors will be installed on the CT equipment to obtain the real-time $I$ data. Third, based on the raw time series, more features should be constructed to reflect more aspects of the status of the equipment. Fourth, currently the parameters such as the window sizes and time lags are obtained by experiments. In the future work, the parameters could be systematically updated to improve the performance of the model.

## Conclusions

The reliability of large-scale medical equipment has been a concern of hospitals and medical institutions. In this study, we propose a novel multivariate time-series classification model that uses the status data from the IoMT to predict the CT equipment anomalies. The statistics and transformations of the raw historical time-series data segment in the sliding time window are used to construct new features. The proposed two frameworks for training and test datasets construction overcome the issues of data imbalance. Of the 5 classification models used, NB has the best performance with the Acc and Rec of 0.79 and 0.77 respectively, which shows the applicability and practicability of predicting medical equipment anomalies based on IoMT with data-driven models. The identified important features may provide instructions to the equipment operators to ensure the reliability of the medical equipment. It is shown that the proposed model is better than existing models in this application.

## Data Availability

The data that support the findings of this study are available from the West China Hospital of Sichuan University but restrictions apply to the availability of these data, which were used under license for the current study, and so are not publicly available. Data are however available from the authors upon reasonable request and with permission of the West China Hospital of Sichuan University.

## Abbreviations

CT: Computed Tomography
TJC: The Joint Commission
CM: Corrective Maintenance
PvM: Preventive Maintenance
PdM: Predictive Maintenance
IoT: Internet of Things
IoMT: Internet of Medical Things
SVM: Support Vector Machines
EL: Ensemble Learning
DL: Deep Learning
OT: Oil Temperature
AV: Anode Voltage
TST: Cumulative Tube Scanning Time
CE: Cumulative Consumption of the Electrical Energy
AOT: Daily Average Oil Temperature
AAV: Daily Average Anode Voltage
ATST: Daily Average Cumulative Tube Scanning Time
ACE: Daily Average Cumulative Consumption of Electrical Energy
DTST: Daily Tube Scanning Time
DCE: Daily Consumption of Electrical Energy
I: Current
DT: Decision Tree
LR: Logistic Regression
NB: Naive Bayesian
KNN: K-Nearest Neighbor
Acc: Accuracy
Rec: Recall
Pre: Precision
F1: F1-score
ROC: Receiver Operating Characteristic
AUC: Area Under the Curves
TP: True Positive
TN: True Negative
FP: False Positive
FN: False Negative
RFs: Random Forests
